# The Effect of Zn-Al-Hydrotalcites Composited with Calcium Stearate and β-Diketone on the Thermal Stability of PVC

**DOI:** 10.3390/ijms12031756

**Published:** 2011-03-08

**Authors:** Mengliang Tong, Hongyan Chen, Zhanhong Yang, Runjuan Wen

**Affiliations:** 1 Hunan Chemical Vocational and Technical College, Zhuzhou 412004, China; E-Mail: 13973327103@163.com; 2 College of Chemistry and Chemical Engineering, Central South University, Changsha 410083, China; E-Mails: chykn0@163.com (H.C.); wrj20050440076@163.com (R.W.)

**Keywords:** Zn-Al-LDHs, polyvinyl chloride, stabilizer

## Abstract

A clean-route synthesis of Zn-Al-hydrotalcites (Zn-Al-LDHs) using zinc oxide and sodium aluminate solution has been developed. The as-obtained materials were characterized by X-ray diffraction (XRD), Fourier transform infrared spectroscopy (FT-IR), and scanning electron microscopy (SEM). The effects of metal ions at different molar ratios on the performance of hydrotalcites were discussed. The results showed that the Zn-Al-hydrotalcites can be successfully synthesized at three different Zn/Al ratios of 3:1, 2:1 and 1:1. Thermal aging tests of polyvinyl chloride (PVC) mixed with Zn-Al-LDHs, calcium stearate (CaSt_2_) and β-diketone were carried out in a thermal aging test box by observing the color change. The results showed that Zn-Al-LDHs can not only enhance the stability of PVC significantly due to the improved capacity of HCl-adsorption but also increase the initial stability and ensure good-initial coloring due to the presence of the Zn element. The effects of various amounts of Zn-Al-LDHs, CaSt_2_ and β-diketone on the thermal stability of PVC were discussed. The optimum composition was determined to be 0.1 g Zn-Al-LDHs, 0.15 g CaSt_2_ and 0.25 g β-diketone in 5 g PVC.

## 1. Introduction

Chlorine-containing polymers such as polyvinyl chloride (PVC) are widely used in many fields, namely, in water pipes, floor and roof tiles, packing films and sheets, due to their advantages of a non-flammable nature, good performance and low price [1]. However, under the influence of elevated temperature and UV radiation, PVC undergoes an autocatalytic dehydrochlorination reaction, which results in severe discoloration and loss of some properties [2]. Stabilizers can inhibit the degradation of removal of HCl due to their capacity for HCl-adsorption. Although the main used thermal stabilizers are lead salts, metal soaps, organo-tin compounds and rare earth compounds [3–4], these stabilizers have some drawbacks of toxicity, environmental pollution and high cost. Hence, it is necessary to explore an environmentally friendly stabilizer with non-toxicity, economical interest and high efficiency.

Layered double hydroxides (LDHs) are a class of synthetic two-dimensional nanostructured anionic clays with a hydrotalcite (HT)-like structure [5]. LDHs are represented by the general formula [M^2+^_1–_*_x_*M ^3+^*_x_*(OH)_2_]*^x^*^+^(A*^n^*^−^)*_x/n_* *m*H_2_O, where M^2+^ (M = e.g., Mg, Fe, Co, Cu, Ni, or Zn) and M^3+^ (M = e.g., Al, Cr, Ga, Mn or Fe) are di-and trivalent cations, respectively; the value of *x* is equal to the molar ratio of M^2+^*/*(M^2+^ + M^3+^), which is generally in the range of 0.2–0.33; A*^n^*^−^ is an anion. Substitution of divalent cations by trivalent cations can result in a positive charge on the layers. So organic or inorganic anions are intercalated between the layers in order to maintain charge balance, and the water of crystallization is also generally found in the interlayer galleries [6–7]. There are many techniques for the preparation of LDHs. The most commonly used method is simple co-precipitation, some other methods, such as rehydration, hydrothermal reaction, pre-pillaring and intercalation, were also reported [8]. Due to their acidic and alkaline characteristics, memory effect, large surface area, high anion exchange and good thermal stability, LDHs present a good prospect for the application in catalysis, ion-exchange, adsorption, pharmaceutics, photochemistry and electrochemistry [9]. Moreover, the largest application of LDHs materials is in the polymer industry, mainly related to stabilizing PVC [10–12].

Mg-Al-CO_3_-LDHs have been reported to incorporate into PVC matrices with other organic salt stabilizers, such as Ca, Zn and Sn stearates, which can improve the thermal stability of PVC significantly [13–14].However, because of the introduction of Mg, the PVC samples with Mg-Al-LDHs have much weaker transparency resulting in yellowish appearance, which cannot meet the demands of plastic in the food industry, where colorless and odorless products are needed. Though this problem can be alleviated with the addition of zinc stearate to a certain degree, it was not resolved fundamentally. In order to achieve a product with initial thermal stability and transparency, the addition of Mg element was forbidden and Mg element should be replaced by Zn element [15].

Co-precipitation is the most common method used in the fabrication of LDHs. However, the products prepared showed low crystalline and fine particles, which needed further complex and various treatments of aging, hydrothermal reaction and so on. In addition, NaOH was consumed vastly, which can react with Cl^−^, NO_3_^−^ and SO_4_^−^ in the raw material forming NaCl, NaNO_3_ and Na_2_SO_4,_which then can be released into the environment in large quantity.

In this work, the hydrotalcites were synthesized by a clean-route using zinc oxide and sodium aluminate solution. When replacing ZnCl_2_, Zn (NO_3_)_2_, ZnSO_4_ with ZnO, zero release of NaCl, NaNO_3_ and Na_2_SO_4_ was achieved. The effects of various molar ratios of Zn/Al on the synthesis of Zn-Al-LDHs were studied. Moreover, the synergistic effect of LDHs, metal soap (CaSt_2_) and β-diketones were discussed in detail.

## 2. Results and Discussion

### 2.1. Characterization of the Zn-Al-LDHs

#### 2.1.1. X-ray Diffraction (XRD) Analysis of Zn-Al-LDHs

**Figure 1.**
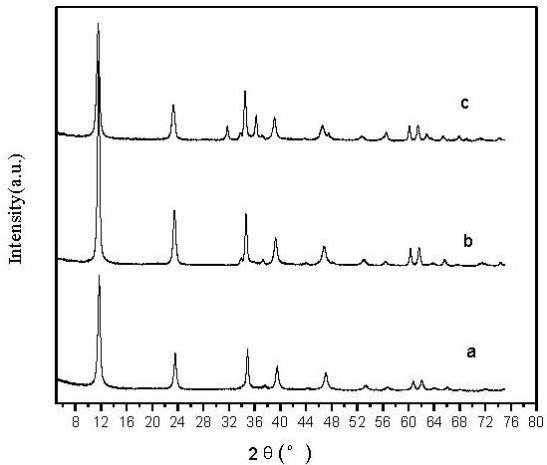
(**a**) The XRD patterns of Zn-Al-LDHs with Zn/Al molar ratios of 1:1; (**b**) 2:1; and (**c**) 3:1.

The XRD patterns of Zn-Al-LDHs with different Zn-Al molar ratios are exhibited in Figure 1. The strong peaks at 11.2°, 23.5°, 35.1°, 61.8°are characteristic of a layered structure. The sharp and symmetrical diffraction peaks with a low and stable baseline indicate the well-crystallized layered structure of the Zn-Al-LDHs. It can be found that the XRD patterns of Zn-Al-LDHs with different Zn/Al molar ratios are similar. The LDHs can be successfully synthesized with three different Zn/Al ratios of 3:1, 2:1 and 1:1. According to *a* = 2d_110_, the crystal structure of Zn-Al-LDHs with different molar ratios are the same. The charge number of Al^3+^ is greater than that of Zn^2+^, hence, the distance of the layers increases and the coulomb repulsion between metal ions in the adjacent hexagonal unit cells decreases with increasing the Zn/Al ratio, making the lattice parameter *a* and *c* (=3d_003_) increase, especially *c*. This is based on the fact that parameter *a* is a function of the average radius of the metal cations while parameter *c* is a function of the average charge of the metal cations [14]. From Figure 1 the diffraction peak of curve b is more sharp than that of a and c, indicating that the Zn-Al-LDHs has high crystallinity and integrated crystalline structures with molar ratio of 2:1.

#### 2.1.2. Fourier Transform Infrared Spectroscopy (FT-IR) Analysis of Zn-Al-LDHs

**Figure 2.**
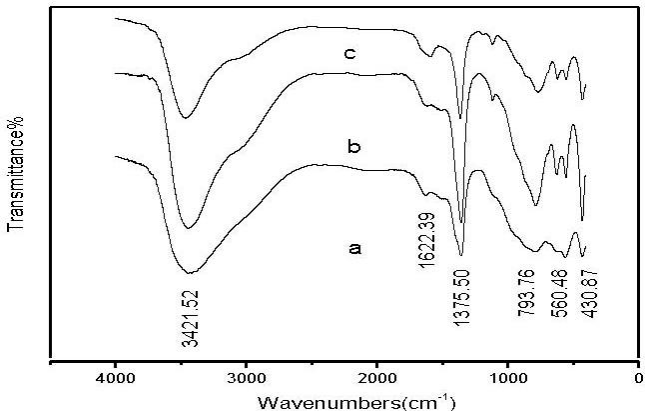
Fourier transform infrared spectroscopy (FT-IR) spectra of Zn-Al-LDHs with Zn/Al molar ratios of (**a**) 1:1; (**b**) 2:1; and (**c**) 3:1.

FT-IR spectra of the LDHs samples are shown in Figure 2. As shown in Figure 2, a band near 3,420 cm^−1^ corresponds to the vibration bands of hydroxyls (νOH). The peak moves to a higher wavenumber with increasing Zn/Al ratio. The possible reason is that the ionic radius of Al^3+^ is smaller than that of Zn^2+^ and the charge number of Al^3+^ is greater than that of Zn^2+^, the electrostatic force between Al^3+^ and OH^−^ is stronger than that of Zn^2+^ and OH^−^ with increasing the Zn/Al ratio. The bending mode of water molecules appears at 1,620 cm^−1^ and the intensity increases slightly with increasing Zn/Al ratio. The sharp intense band at 1,364 cm^−1^ is the antisymmetric stretching of interlayer carbonate with high values of Zn/Al ratio. Compared with the peak of CO_3_^2−^ in CaCO3 (1,430 cm^−1^), a large shift toward a lower wavenumber indicates that the CO_3_^2−^ intercalating into the interlayer is not free, and is interlayered with water molecules between with hydrogen bonds are formed [16]. The interlayer charge density and the coulomb repulsion between interlayer anions increases with decreasing Zn/Al ratio, which results in a stronger peak of interlayer CO_3_^2−^ and a lower wave number (1,357 cm^−1^). The band at 794 cm^−1^ is the flexural vibration of CO_3_^2−^. The bands at 430 cm^−1^ and 560 cm^−1^ can be attributed to the lattice vibration modes, M-O and O-M-O vibration, respectively. The structural parameters of FT-IR are listed in Table 1.

#### 2.1.3. Scanning Electron Microscope (SEM) Micrograph of Zn-Al-LDHs

**Figure 3.**
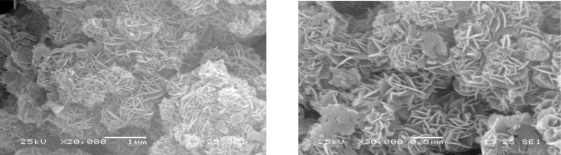
Two examples of scanning electron microscope (SEM) images of Zn-Al-LDHs with a Zn-Al molar ratio of 3:1.

Figure 3 shows typical SEM micrographs of Zn-Al-LDHs with a Zn-Al molar ratio of 3:1. It can be seen that the existing lamellar particles have a rounded hexagonal shape, which is the typical structure of the hydrotalcite-like materials.

#### 2.1.4. Particle Granularity and Specific Area Analysis of Zn-Al-LDHs

**Figure 4.**
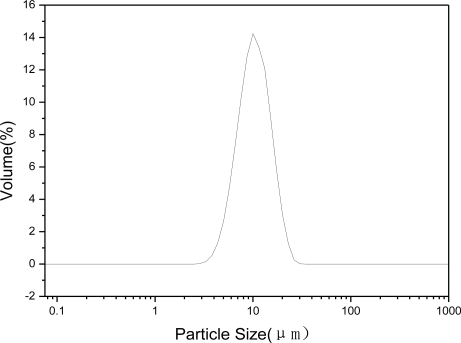
Particle granularity analysis pattern of Zn-Al-hydrotalcite.

Figure 4 shows the particle granularity analysis pattern of Zn-Al-LDHs with a Zn-Al molar ratio of 3:1. The range of the particle granularity was relatively narrow and the particle size showed normal distribution, which indicates the particle size distribution of powders was homogeneous and narrow.

The specific area of Zn-Al-LDHs with Zn-Al ratio of 3:1 was analyzed by adsorption apparatus. The specific area of the particles is 43.48 m^2^/g and the pore volume is 0.2613 cm^3^/g.

### 2.2. Synergetic Thermal Stabilizing Effect of Zn-Al-LDHs with Metal Soap and β-Diketone

5 g PVC, 2.5 g DOP, and various amounts of Zn-Al-LDHs, Cast_2_ and β-diketone were mixed. The results of static thermal aging tests carried out at 180 ± 1 °C are shown in Table 2, 3 and 4. The thermal stability of PVC is defined by the time taken until black composition appeared. The β-diketone acts as a short-term stabilizer and the Zn-Al-LDHs acted not only as a long-term stabilizer because of hydroxyl, but also a short-term one due to Zn^2+^. However, with high content of Zn, the performance of long-term stabilizer was weakened, and then CaSt_2_, a long-term stabilizer, was used to cooperate with Zn-Al-LDHs and β-diketone, which can enhance the stability of PVC greatly.

#### 2.2.1. The Effect of Zn-Al-LDHs, CaSt_2_ and β-Diketone on the Thermal Stability of PVC

**Figure 5.**
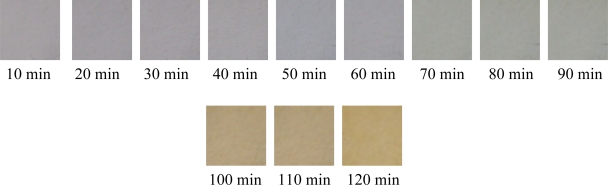
The color stability of PVC with 0.1 g LDHs, 0.2 g CaSt_2_ and 0.25 g β-diketone.

As shown in Table 2, PVC mixture without Zn-Al-LDHs (serial number 1) became completely black within just 10 min at 180 ± 1 °C. When 0.04 g LDHs (serial number 2) was added to the PVC, the stability time increases to 70 min, indicating that the thermal stability is significantly enhanced. The reaction between Zn-Al-CO_3_-LDHs and HCl occurs in a two-step process. First of all, the counter ions OH^−1^ and CO_3_^2−^ between the LDH layers react with the HCl gas. Then, the LDH itself reacts with HCl with complete destruction of the LDH structure and formation of metal chlorides. The Zn-Al-CO_3_-LDHs can react with labile chlorine atoms (such as allyl chloride), so it is easy to restrain the initial coloring and prevent the growth of “Polyolefin Structure” when it is used alone. As the amount of LDHs increased from 0 to 0.1 g, there is a prominent increase in thermal stability of PVC. However, further addition of LDHs in PVC cannot enhance the thermal stability more, and weakens the stability instead. This phenomenon can be illustrated as the quantities of Zn increasing with the increment of LDHs, leading to the formation of ZnCl_2_ which can catalyze the dehydrochlorination. In Zn-Al-LDHs, hydroxyl can absorb HCl; furthermore, the existance of Zn can increase the initial stability, which makes good-initial coloring. However, the long-term stability is impaired due to the “zinc burning” as adding excess Zn-Al-LDHs. Compared with Mg-Al-LDHs, the PVC samples with Zn-Al-LDHs are light yellow after 110 min in the aging test in this work, while the samples with Mg-Al-LDHs turned yellow inclining to black during the initial 20 min [17]. The optimum amount of LDHs is determined to be 0.1 g from Table 2. Xu *et al*. [18] investigated the thermal stability of Zn-Al-CO_3_-LDH-incorporated PVC at high temperatures and found that LDHs facilitated dehydrochlorination at a lower temperature but LDHs can reduce the subsequent dehydrocarbonation and help to form more char-like carbonaceous materials. Unlike that investigation, the CaSt_2_ and β-diketone added here cooperated with LDHs to effectively reduce the formation of ZnCl_2,_ then the dehydrochlorination of HCl was impaired even at initialization. The photos for the color changes of PVC with 0.1 g LDHs are displayed in Figure 5.

#### 2.2.2. The Effect of Varying the Amount of CaSt_2_ on the PVC

Table 3 shows the color changes of PVC with various amounts of CaSt_2_ added. It is clear that the short-term stabilization of PVC is enhanced with increasing quantities of CaSt_2_. The CaSt_2_ not only absorbs HCl, but also exchanges ions with ZnCl_2_ in the PVC-Zn-Al-LDHs systems, which could be expressed as following:

$${\text{ZnCl}}_{2}\;+\;\text{Ca}{\left(\text{OOCR}\right)}_{2}\;\to \;\text{Zn}{\left(\text{OOCR}\right)}_{2}\;+\;{\text{CaCl}}_{2}$$

**Figure 6.**
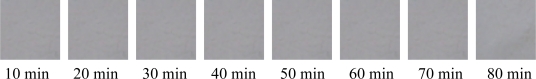

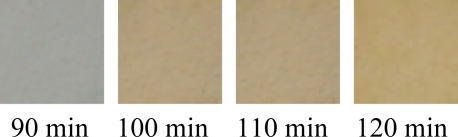
The color stability of PVC with 0.1 g LDHs, 0.15 g CaSt_2_ and 0.25 g β-diketone.

The active ZnSt_2_ is generated and the undesirable ZnCl_2_ is consumed. The product ZnSt_2_ can scavenge HCl and react with the labile chlorine (allylic chlorine) atoms on the PVC chain. From Table 3, the optimum amount of CaSt_2_ is thought to be 0.15 g. The photos for the color changes of PVC with 0.15 g CaSt_2_ are presented in Figure 6.

#### 2.2.3. The Effect of Varying the Amount of β-Diketone on the PVC

**Figure 7.**
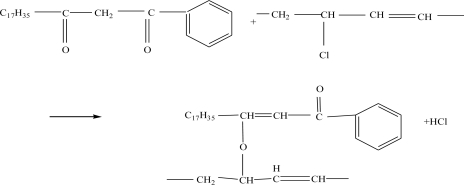
The reaction of β-Diketone with polyvinyl chloride.

The color stability of PVC with β-Diketone is shown in Table 4. For PVC mixtures without any β-Diketone or with a low amount of β-Diketone (series 4 and 5), the discoloration time is only 10 min. The later color change from yellow to black is sudden. When the quantity of β-diketone is 0.25 g, the initial color is stable to some degree, as well as the later color, which means that the optimum amount of β-Diketone is 0.25 g. The β-diketone behaves as a short-term co-stabilizer and increases initial color stability. The acetylacetonates substitute allylic chlorides inhibiting the formation of long polyenes and resulting in no discoloration of the polymer [19].The reaction of β-Diketone with polyvinyl chloride is presented in Figure 7.

**Figure 8.**
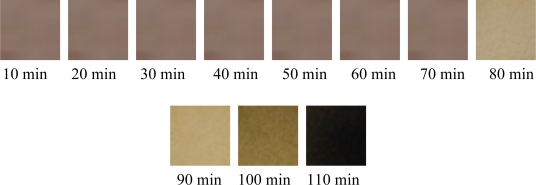
The color stability of PVC with 0.1 g LDHs, 0.15 g CaSt_2_ and 0 g β-diketone.

The photos for the color changes of PVC without β-diketone are shown in Figure 8.

## 3. Experimental Section

### 3.1. Materials

ZnO, NaAlO_2_, Na_2_CO_3_, NaOH, β-diketones, CaSt_2_ and DOP were of analytical grade. The PVC resin (SG-3) used in the experiment was provided by Beijing Huaer.

### 3.2. Synthesis of Zn-Al-CO_3_-LDHs

A NaAlO_2_ solution was added slowly to the mixed solution containing ZnO and Na_2_CO_3_ at 90 °C. The resulting solution was continuously stirred for 2 hours and the pH was adjusted from 9 to 10 using NaOH solution. Subsequently, the solution was maintained at 100 °C for 16 hours in an autoclave. Subsequently, the slurry was aged at 80 °C for 8 hours. Finally, the precipitate was filtered, washed and dried.

### 3.3. Characterization Techniques for Hydrotalcites

Powder XRD patterns were recorded using a Rigaku D/max-2500 diffractometer (Cu Kα radiation, scan speed of 8°/min). Infra red spectra were performed on a Nicolet Nexus-670 FT-IR spectrometer in the range 4000–400 cm^−1^using KBr pellets. The surface morphology of synthesized hydrotalcite was observed using a JSM-6360 scanning electron microscope. The specific area of Zn-Al-LDHs was determined on TriStar3000 BET surface area measurement. The particle granularity was recorded by a laser particle analyzer.

### 3.4. Static Thermal Aging Test

Mixtures containing 5 g PVC powder, 2.5 g dioctylphthalate (DOP), various amounts of LDHs, CaSt_2_ and β-diketone were blended in a double-roller mixer for 5 mins at 180 °C. The resulting composites were molded to sheets with a thickness of 1.0 mm and cut into 3 cm × 2 cm strips. These strips were placed in a Thermal Aging Test Box at 180 ± l °C and subjected to static thermal aging. The strips were taken out of the box every 10 or 20 mins and examined visually.

## 4. Conclusions

In this research, the Zn-Al-CO_3_-LDHs were prepared by a clean-route synthesis method. The hydrotalcite has a layered structure. The LDHs can be successfully synthesized at three different ratios of 3:1, 2:1 and 1:1 and the Zn-Al-LDHs have high crystalline and integrated crystalline structures with Zn/Al molar ratio of 2:1. The Zn-Al-CO_3_-LDHs can react with labile chlorine atoms, so it is easy to restrain the initial coloring and prevent the growth of “Polyolefin Structure” when it is used alone. The Zn-Al-CO_3_-LDHs bear a synergistic effect with CaSt_2_ and β-diketone on the stability of PVC with the optimum quantity of 0.1 g LDHs, 0.15 g CaSt_2_ and 0.25 g β-diketone in 5 g PVC.

## Figures and Tables

**Table 1.** Fourier transform infrared spectroscopy (FT-IR) structure parameters of samples synthesized with different Zn/Al molar ratios.

**molar ratio**	**Wavenumber/cm^−1^**				
	**vibration bands of –OH**	**Antisymmetric stretching of CO_3_^2−^**	**flexural vibration of CO_3_^2−^**	**lattice vibration of (Zn^2+^ Al^3+^)**	
1:1	3421	1357	793	560	430
2:1	3446	1357	785	555	429
3:1	3462	1364	768	553	430

**Table 2.** The color changes for polyvinyl chloride (PVC) with different contents of LDHs, 0.2 g CaSt_2_ and 0.25 g β-diketone.

**Serial number**	**LDHss/g**	**Time/min and color stability**						
		**10**	**30**	**50**	**70**	**90**	**100**	**110**
1	0	7	7	7	7	7	7	7
2	0.04	0	1	3	4	5	7	7
3	0.06	0	1	3	4	5	5	7
4	0.08	0	0	0	3	4	5	6
5	0.1	0	0	0	1	1	3	3
6	0.2	0	0	1	4	5	5	6
7	0.3	0	3	3	4	5	6	6

Color code: 0—white; 1—milky; 2—pink; 3—light yellow; 4—yellow; 5—black and yellow; 6—brown; 7—black.

**Table 3.** The color changes for PVC with different contents of CaSt_2_, 0.1 g LDHs, and 0.25 g β-Diketone.

**Serial number**	**CaSt_2_/g**	**Time/min and color stability**						
		**10**	**30**	**50**	**70**	**90**	**100**	**110**
1	0.45	0	1	1	3	4	4	4
2	0.35	0	1	1	3	3	3	7
3	0.25	0	1	1	3	4	4	6
4	0.2	0	0	0	1	1	3	3
5	0.15	0	0	0	0	1	3	3
6	0.1	0	0	1	3	3	4	5
7	0.05	0	0	1	3	6	7	7

Color code: 0—white; 1—milky; 2—pink; 3—light yellow; 4—yellow; 5—black and yellow; 6—brown; 7—black.

**Table 4.** The color changes observed for PVC with different contents of β-diketone, 0.1 g LDHs and 0.15 g CaSt_2_.

**Serial number**	**β-Diketone/g**	**Time/min and color stability**						
		**10**	**30**	**50**	**70**	**90**	**100**	**110**
1	0.35	0	1	3	3	5	7	7
2	0.25	0	0	0	0	1	3	3
3	0.15	0	0	1	3	4	5	6
4	0.05	1	1	2	2	3	4	7
5	0	2	2	2	2	4	6	7

Color code: 0—white; 1—milky; 2—pink; 3—light yellow; 4—yellow; 5—black and yellow; 6—brown; 7—black.
